# Supplementary material for: Gold Nanoclusters: Bridging Gold Complexes and Plasmonic Nanoparticles in Photophysical Properties
Source: Nanomaterials (Basel). 2019 Jun 28;9(7):933. doi: 10.3390/nano9070933 (PMC6669669; doi:10.3390/nano9070933)
Supplement: Supplementary file 1 [file nanomaterials-09-00933-s001.pdf]

Supplementary Materials:

Gold Nanoclusters: Bridging Gold Complexes and Plasmonic Nanoparticles in Photophysical Properties

Meng Zhou, Chenjie Zeng, Qi Li, Tatsuya Higaki and Rongchao Jin *

Department of Chemistry, Carnegie Mellon University, Pittsburgh, Pennsylvania 15213, USA; mxz572@miami.edu (M.Z.); chenjiez@sas.upenn.edu (C.Z.); qilistan@stanford.edu (Q.L.); thigaki@andrew.cmu.edu (T.H.)

* Correspondence: rongchao@andrew.cmu.edu

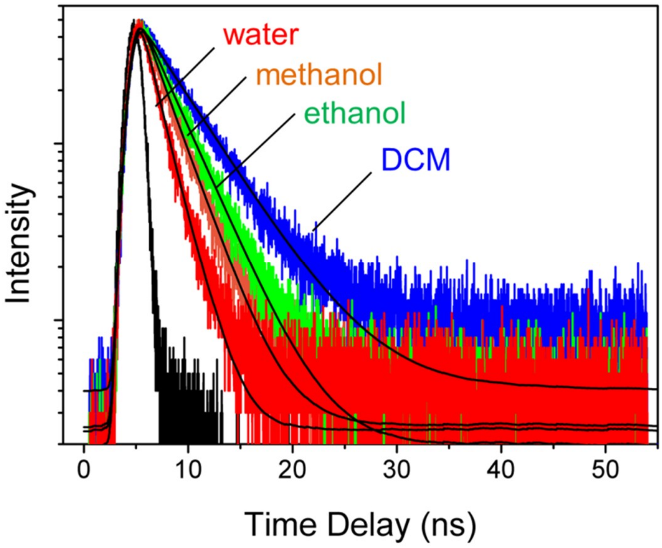

Figure S1. Time resolved photoluminescence of Rhodamine B dissolved in different solvents measure under magic angle (54.7°).

Table S1. Photoluminescence decay lifetime of Rhodamine B dissolved in different solvents.

	Dielectric Constant	τ (ns)
Water	80	1.7
Methanol	32.7	2.5
Ethanol	24.5	3.1
Dichloromethane	8.93	4.6

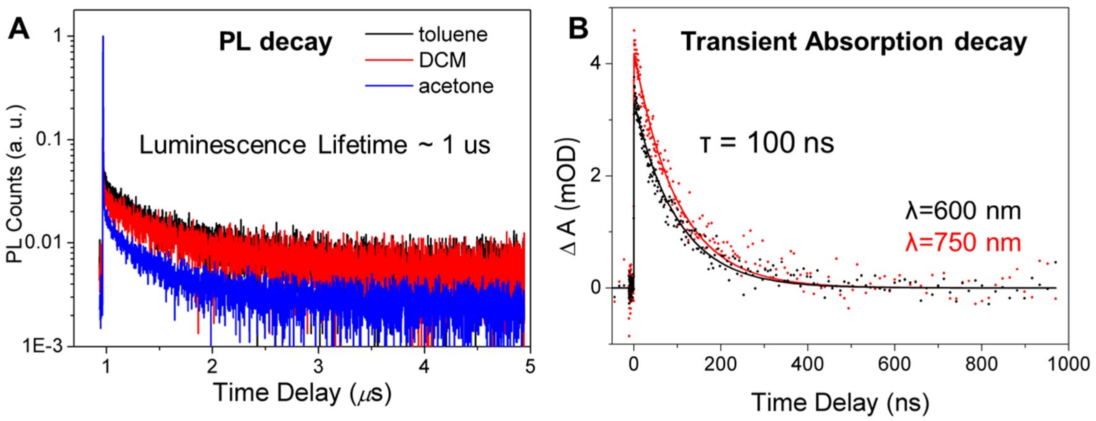

Figure S2. (A) Photoluminescence decay of $Au_{25}(SR)_{18}$ probed at 750 nm in different solvents; (B) Transient absorption decay probed at 600 nm and 750 nm. It is found that TA decays much faster than PL.

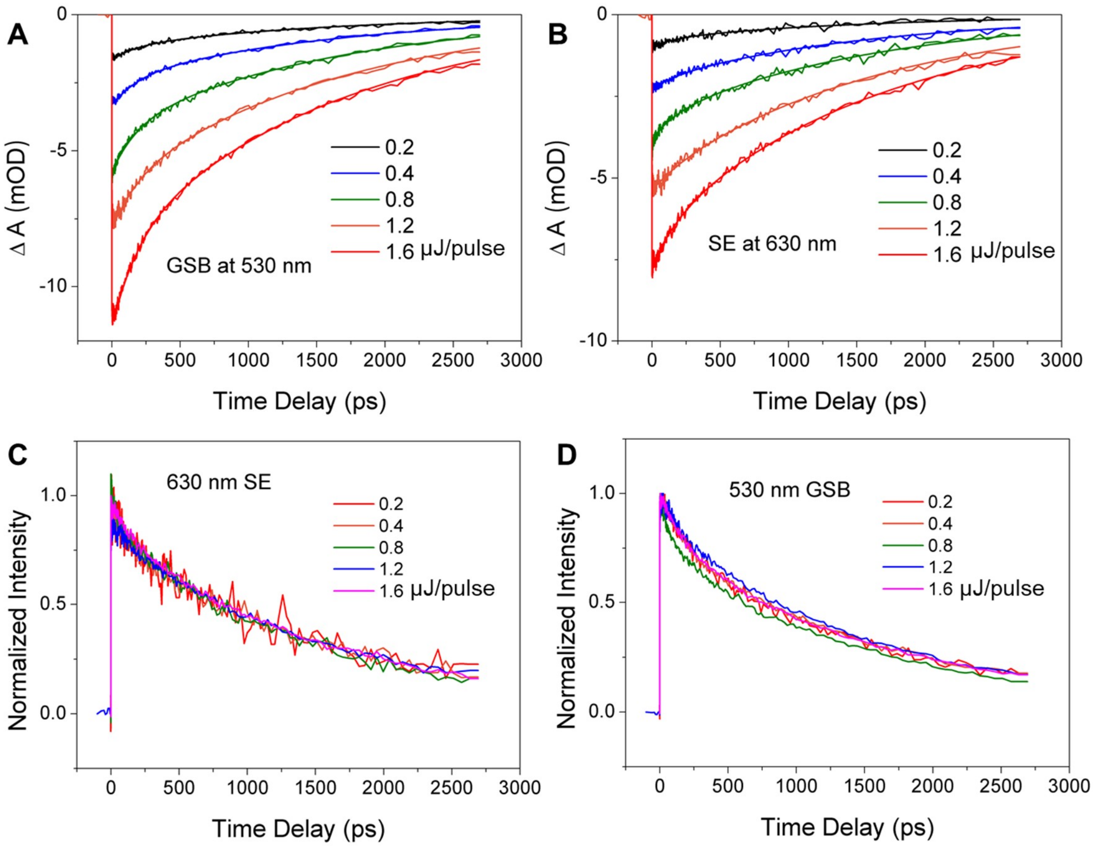

Figure S3. (A-B) Transient absorption decay of Rhodamine B probed at GSB and SE as a function of pump power with 560 nm excitation; (C-D) Normalized decay as a function of pump power.

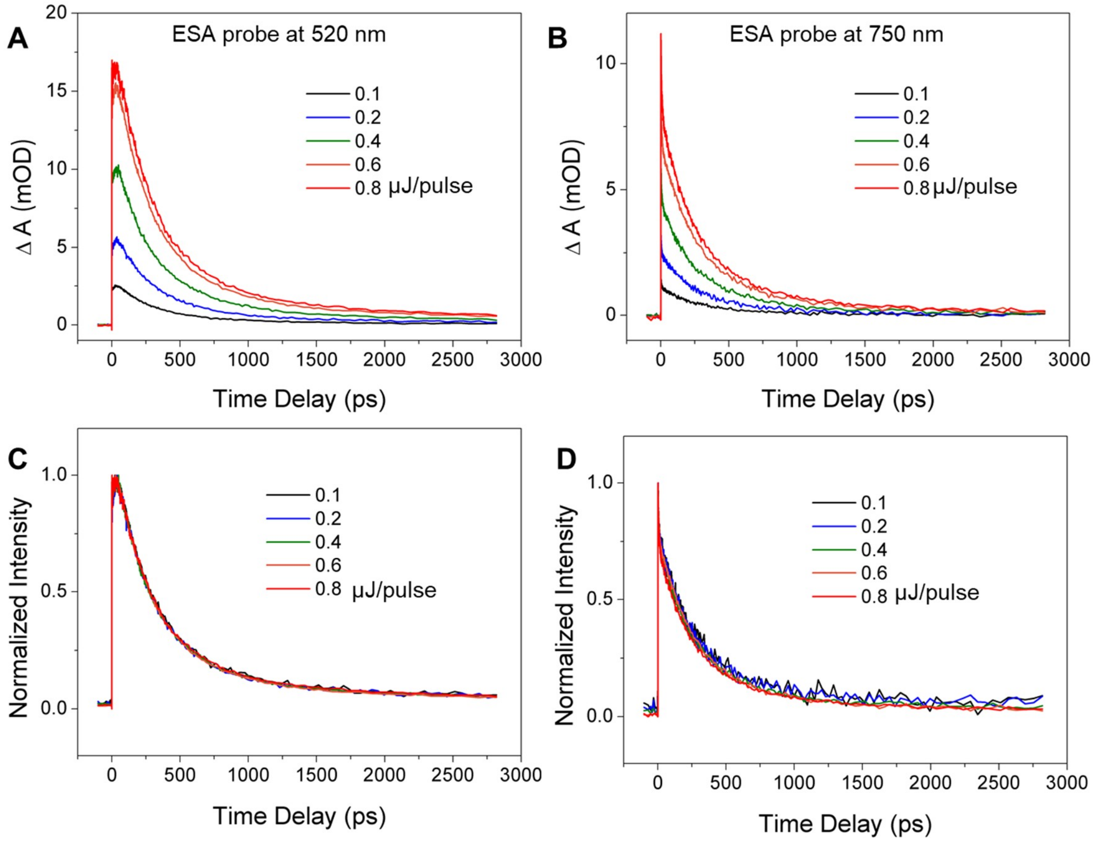

Figure S4. (A-B) Transient absorption decay of $Au_{10}(SR)_{10}$ complexes probed at ESA around 520 nm and 750 nm as a function of pump power with 360 nm excitation; (C-D) Normalized decay as a function of pump power.

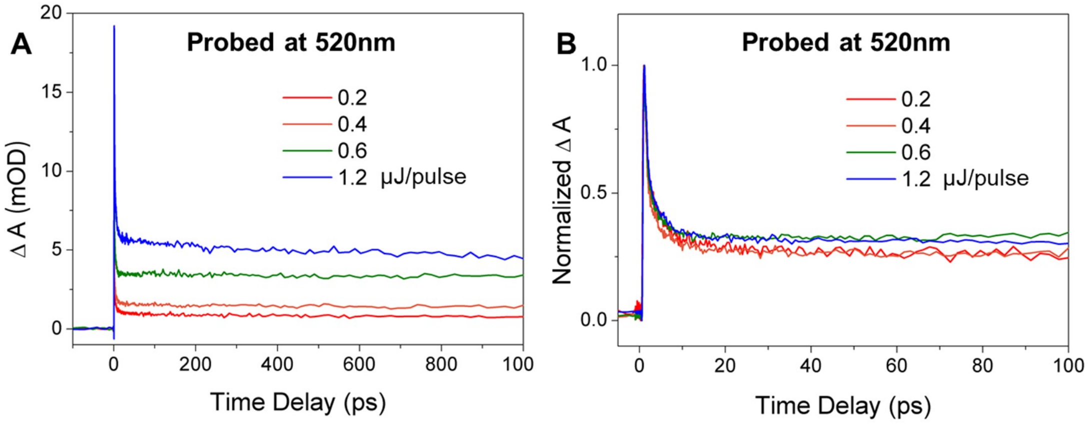

Figure S5. (A) Transient absorption decay of $Au_{10}(SR)_{10}$ complexes probed at ESA around 520 nm as a function of pump power with 490 nm excitation; (B) Normalized decay as a function of pump power.
